# Reconsidering the role of blood-brain barrier in Alzheimer’s disease: From delivery to target

**DOI:** 10.3389/fnagi.2023.1102809

**Published:** 2023-02-16

**Authors:** João André Sousa, Catarina Bernardes, Sara Bernardo-Castro, Miguel Lino, Inês Albino, Lino Ferreira, José Brás, Rita Guerreiro, Miguel Tábuas-Pereira, Inês Baldeiras, Isabel Santana, João Sargento-Freitas

**Affiliations:** ^1^Department of Neurology, Centro Hospitalar e Universitário de Coimbra, Coimbra, Portugal; ^2^Faculty of Medicine, University of Coimbra, Coimbra, Portugal; ^3^Centre for Neuroscience and Cell Biology, University of Coimbra, Coimbra, Portugal; ^4^Department of Neurodegenerative Science, Van Andel Institute, Grand Rapids, MI, United States; ^5^Centre for Innovative Biomedicine and Biotechnology (CIBB), University of Coimbra, Coimbra, Portugal

**Keywords:** blood-brain barrier, nanomaterials, Alzheimer’s disease, neurodegenerative diseases, amyloid clearance, genetic targeting

## Abstract

The existence of a selective blood-brain barrier (BBB) and neurovascular coupling are two unique central nervous system vasculature features that result in an intimate relationship between neurons, glia, and blood vessels. This leads to a significant pathophysiological overlap between neurodegenerative and cerebrovascular diseases. Alzheimer’s disease (AD) is the most prevalent neurodegenerative disease whose pathogenesis is still to be unveiled but has mostly been explored under the light of the amyloid-cascade hypothesis. Either as a trigger, bystander, or consequence of neurodegeneration, vascular dysfunction is an early component of the pathological conundrum of AD. The anatomical and functional substrate of this neurovascular degeneration is the BBB, a dynamic and semi-permeable interface between blood and the central nervous system that has consistently been shown to be defective. Several molecular and genetic changes have been demonstrated to mediate vascular dysfunction and BBB disruption in AD. The isoform ε4 of Apolipoprotein E is at the same time the strongest genetic risk factor for AD and a known promoter of BBB dysfunction. Low-density lipoprotein receptor–related protein 1 (LRP-1), P-glycoprotein, and receptor for advanced glycation end products (RAGE) are examples of BBB transporters implicated in its pathogenesis due to their role in the trafficking of amyloid-β. This disease is currently devoid of strategies that change the natural course of this burdening illness. This unsuccess may partly be explained by our misunderstanding of the disease pathogenesis and our inability to develop drugs that are effectively delivered to the brain. BBB may represent a therapeutic opportunity as a target itself or as a therapeutic vehicle. In this review, we aim to explore the role of BBB in the pathogenesis of AD including the genetic background and detail how it can be targeted in future therapeutic research.

## 1. Introduction

Neural activity and blood flow are intertwined in a relationship that can be summarized as neurovascular coupling. The immediate increase in cerebral blood flow allows for prompt oxygen and glucose delivery to brain areas that are selectively being activated (Freygang and Sokoloff, 1959; Cox et al., 1993; Chaigneau et al., 2003). This unique and sophisticated mechanism compensates for the lack of energy reserves in the brain (Iadecola, 2017). Whenever there is an imbalance between the energy demands of neural tissue and the blood delivery either a complete flow interruption (such as in stroke) or a more subtle and chronic insufficiency, there is a brain injury and cognitive impairment (Iadecola, 2013, 2017). Deficient blood flow may also lead to the accumulation of toxic waste products such as amyloid β (Aβ) and tau (Tarasoff-Conway et al., 2015; Iadecola, 2017). The Italian physiologist Mosso first showed this intimate relationship between blood and brain activity in 1878. He demonstrated that brain pulsations in the right prefrontal cortex of a subject with a skull defect rose when he performed an arithmetic task (Raichle and Mintun, 2006). One century later, Lassen et al. (1978) showed an increase in cerebral blood flow in the contralateral sensory-motor cortex and the supplementary motor area produced by hand movement, heralding the functional brain imaging era.

We now know that at the crossroads of brain and blood, there is the neurovascular unit (NVU), a concept formalized in 2001 amongst the first Stroke Progress Review Group meeting of the National Institute of Neurological Disorders and Stroke of the National Institute of Health. NVU offered a view that was opposed to the rigid separation between neurodegenerative diseases, the commonest being Alzheimer’s disease (AD), and cerebrovascular diseases (Iadecola, 2017).

The anatomical substrate of this functional brain-vascular unit is the blood-brain barrier (BBB) which was described first-hand by Paul Elrich in 1885 (Khatri et al., 2012) and Dyrna et al. (2013). The neurovascular contact changes throughout the cerebrovascular tree. On the pial surface, there is a thick vascular muscle cell layer, subarachnoid space, and autonomic and sensory nerve fibers around the pial arterioles. Penetrating arterioles enter the parenchyma but are separated from it by a perivascular space. In the parenchyma and at a capillary level, perivascular space disappears, and pericytes and basement membrane surround endothelial cells forming the BBB as we know it (Iadecola, 2017).

The BBB is a dynamic interface that regulates the cellular communication between neural tissues and the blood and its constituents. It acts as a selective semipermeable barrier that controls the transport of substances to and from the central nervous system, serving as a key player in neural homeostasis. The blood is separated from CNS by brain endothelial cells separated by tight junctions, adherens junctions, and gap junctions; pericytes; the foot processes of astrocytes, and the basement membrane composed of extracellular matrix components. Two main transport pathways occur within BBB: transcellular *via* endothelial used by the vast majority of the molecules which can be active (dependent on energy) or passive; and paracellular *via* passive diffusion through tight junctions.

The ubiquity and importance of BBB in CNS physiology also translate to how it is also impaired in almost every neurological condition. Such is the case of the most common cause of dementia, AD. AD affects more than 30 million people worldwide, a number that is expected to increase dramatically in the foreseeable future (Winblad et al., 2016). To date, intracellular hyper-phosphorylated tau protein accumulation (neurofibrillary tangles) and extracellular Aβ deposition (senile plaques) in brain parenchyma is considered the central neuropathological hallmarks of the disease (Selkoe and Hardy, 2016). However, pathogenesis is still not fully understood, and it is unclear whether these protein abnormalities are causative or rather incidental changes in the disease. Nevertheless, it is generally accepted that both proteins play a key role in disease pathogenesis with Aβ acting upstream of tau (Bloom, 2014) with other hypotheses building on and extending this to explain other aspects of the disease (Calsolaro and Edison, 2016; De Strooper and Karran, 2016; Goetzl and Miller, 2017).

Aβ deposition seems to be a critical pathological trigger in AD and disruption of BBB leads to increased vascular permeability, allowing the entrance and/or hampering the clearance of toxic molecules that can trigger inflammatory and immune responses and, ultimately, neurodegeneration (Iadecola, 2013; Kisler et al., 2017; van Dyck, 2018). One such pathologic protein whose normal clearance is dependent on a healthy BBB is the 42 amino acid Aβ peptide (Aβ42), considered the major toxic Aβ in AD. Not surprisingly, BBB dysfunction leads to Aβ deposition by disrupting its transporters (Huang et al., 2020; Wang et al., 2021). Moreover, there is experimental evidence that a disrupted BBB promotes its production from the amyloid precursor protein (APP) through the activation of the amyloidogenic pathway where APP is cleaved in sequence by β-and γ-secretase (Ridler, 2018; Wang et al., 2018).

**FIGURE 1:**
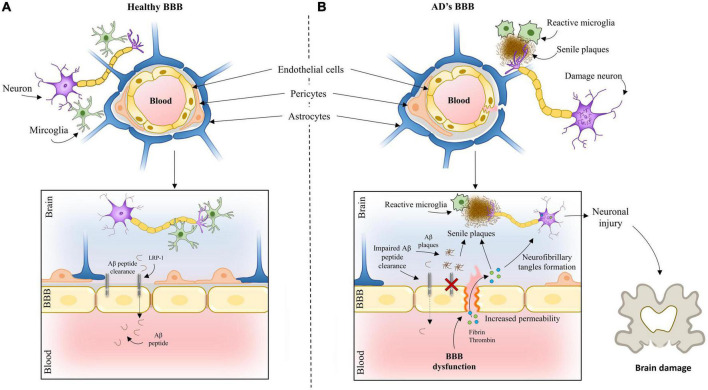
The blood-brain barrier in an **(A)** healthy brain and **(B)** Alzheimer’s brain.

Several studies have demonstrated BBB breakdown and dysregulation in AD (Montagne et al., 2017). Figure 1 presents BBB in a healthy brain and AD. Whether it is a cause or consequence of the disease has been a matter of debate. Available evidence points to BBB breakdown as an early event preceding AD pathology (Iturria-Medina et al., 2016). These findings have been supporting the vascular hypothesis of AD. First published in 1993 by de la Torre et al. this hypothesis postulates that neurodegeneration is the consequence of a series of pathogenic pathways originating in blood vessels (de la Torre and Mussivan, 1993). More recently, Zlokovic (2011) proposed the two-hit vascular hypothesis of AD. According to this hypothesis, impairment of blood vessels leads to BBB dysfunction and initiates a cascade of events leading to neuronal dysfunction (hit one). BBB dysfunction reduces Aβ clearance and increases its production inducing accumulation of this peptide, amplifying neuronal dysfunction, and accelerating neurodegeneration (hit two).

Blood-brain barrier has been emerging as a central hub for AD pathogenesis, presenting as a potential target to treat AD. Understanding its dysfunctional role in AD pathogenesis would be paramount for AD biology clarification and would probably give insights into other brain disorders.

In this review, we will detail pathogenic and therapeutic links between AD and BBB offering a comprehensive and integrative view that includes the genetic landscape of AD and anticipates future research and treatment.

## 2. Fundamental blood-brain barrier concepts in the context of Alzheimer’s disease

Alzheimer’s disease pathogenesis can be viewed in light of two distinct paradigms: the amyloid-cascade hypothesis and the vascular hypothesis. The first hypothesis states that Aβ deposition is the initial step in AD pathogenesis. Alternatively, the vascular hypothesis states that damage to blood vessels is the initial insult leading to neuronal injury and Aβ accumulation.

The BBB is responsible for the clearance of 80–85% of AD-related forms of Aβ in the brain (Xie et al., 2013). Increasing evidence points to BBB dysfunction as an early biomarker of neurodegeneration, including AD (Montagne et al., 2015; Sweeney et al., 2018). Neuroimaging tools (Protas et al., 2013; Bailly et al., 2015; Montagne et al., 2015), *in vivo* disease animal models (Niwa et al., 2002) and post-mortem human studies (Salloway et al., 2002; Wu et al., 2005; Ryu and McLarnon, 2009; Bell et al., 2010; Hultman et al., 2013; Sengillo et al., 2013; Zenaro et al., 2015; Halliday et al., 2016) have allowed identifying key functional and molecular changes occurring during AD across different regions of the brain. Particularly, the development of advanced brain imaging techniques increased the detectability of vascular changes and hemodynamic responses (Kisler et al., 2017). Molecular ligands, such as Aβ and tau, but also the glucose and P-glycoprotein 1 analog, 18F-fluorodeoxyglucose and 11C-verapamil, allowed to follow the activity of BBB transporters and receptor proteins *in vivo* (Protas et al., 2013; Deo et al., 2014), providing mechanistic insights into the role of vascular dysfunction in neurodegenerative diseases. BBB breakdown was confirmed by dynamic contrast-enhanced MRI studies showing increased leakage of gadolinium in patients with early AD, in several gray and white matter regions (Montagne et al., 2016). Evidence of BBB disruption in AD has also been confirmed by the quantification of the accumulation of blood-derived neurotoxic proteins, such as fibrinogen, thrombin, albumin and IgG in the cortex and hippocampus of post-mortem tissues (Ryu and McLarnon, 2009). Interestingly, often these proteins colocalize with deposits of Aβ. The identification of peripheral macrophages (Hultman et al., 2013) and neutrophils (Zenaro et al., 2015) in the brain of individuals with AD also suggest a breakdown of the BBB, leading to increased influx of peripheral immune cells into the brain. Other findings indicative of endothelial degeneration in AD include reduced capillary length, reduced expression of tight junction proteins and capillary basement membrane changes (Salloway et al., 2002; Halliday et al., 2016). At cellular level, clinical studies have revealed a reduction in pericyte number and coverage in the cortex and hippocampus of AD individuals (Sengillo et al., 2013). Importantly, it has been demonstrated that a deficiency in brain pericytes in murine central nervous system lead to vascular damage through a reduction in brain microcirculation as well as *via* BBB breakdown with toxic extravasation of plasma proteins (Bell et al., 2010).

Several molecular changes have been linked to the vascular dysfunction and disruption of the BBB in AD. Apolipoprotein E (APOE) is one of the genes with a critical role in neurovascular dysfunction and is the strongest genetic risk factor for AD. APOE ε4 homozygotes with AD have thinner capillary basement membranes (Salloway et al., 2002) and increased leakage of plasma proteins into the cortex (Salloway et al., 2002). It has been found that APOE ε4 leads to BBB breakdown, decreased cerebral blood flow, neuronal loss and behavioral deficits independently of Aβ (Montagne et al., 2021). Particularly, the activation of cyclophilin A-matrix metalloproteinase 9 (CypA-MMP-9) pathway in pericytes in APOE ε4 knock-in mice was found to lead to matrix metalloproteinase 9 mediated degradation of tight junction proteins, namely, through ZO-1 and occludin (Montagne et al., 2021). This pathway is also activated at the BBB endothelial cells and pericytes in APOE ε4 AD carriers as shown by post-mortem tissue (Halliday et al., 2016) analyses and cerebrospinal fluid analyses (Montagne et al., 2020).

The pathogenesis of AD has also been associated with a decreased expression of GLUT1, the major glucose transporter in the BBB. Several studies reported that GLUT1 levels are significantly reduced in brain microvessels in AD (Simpson et al., 1994). Moreover, changes in glucose metabolism were shown to occur before neuronal dysfunction in humans (Protas et al., 2013) and in transgenic AD models (Niwa et al., 2002), as evaluated by measuring the radiolabeled glucose analogue, 18F-fluoro-2-deoxyglucose by positron emission tomography. A decreased glucose uptake in the hippocampus, parietal cortex and posterior cingulate cortex has been observed in early AD (Bailly et al., 2015) as well as in individuals at increased genetic risk of AD (Protas et al., 2013). In line with this, it has been demonstrated that a deficiency in this transporter in transgenic mice overexpressing human APP can exacerbate neurovascular dysfunction in AD by generating a series of parallel pathogenic mechanisms in the cerebral microcirculation, namely, a significant reduction in the expression of tight junctions, capillary degeneration, neurovascular uncoupling, BBB breakdown, decreased brain perfusion and impaired Aβ clearance (Winkler et al., 2015). Aberrant expression of other transporters at the BBB has also been implicated in the pathogenesis of AD, such as receptors directly involved in the transport of Aβ across the BBB. This includes low expression of low-density lipoprotein receptor–related protein 1 (LRP-1), a major clearance receptor for Aβ at the BBB (Shibata et al., 2000). Pharmacological inhibition and genetic knock-down of LRP-1 in APP/PS1 mouse model of AD accelerated brain accumulation of Aβ and exacerbated Aβ deposition as amyloid plaques and cerebral amyloid angiopathy without affecting Aβ production (Kanekiyo et al., 2012). Moreover, the metabolism and transport of Aβ across the BBB are mediated differently by the different APOE isoforms through receptor-mediated transcytosis. APOE ε2 and APOE ε3 mediate rapid Aβ clearance by interacting with LRP-1, whereas APOE ε4 has a higher affinity to very low-density lipoprotein receptor (VLDR) resulting in slower clearance of Aβ and increased accumulation in the brain (Deane et al., 2008). P-glycoprotein (P-gp), an ATP-binding cassette transporter highly expressed on the luminal side of the BBB, is involved in the clearance of Aβ from the brain, promoting the export of Aβ in combination with LRP-1. The expression of this transporter is decreased in aged individuals and in AD (Chiu et al., 2015). This decline in protein expression also translates into reduced protein function. PET studies revealed that the clearance of the labeled P-gp substrate (R)-[11C]-verapamil is decreased in individuals with mild to moderate AD (van Assema et al., 2011). Besides LRP-1 and P-gp, the levels of the receptor for advanced glycation end products (RAGE) are also altered in AD (Miller et al., 2008). This receptor transports Aβ in opposite direction to LRP-1 mediating the re-entry of circulating Aβ into the brain and thereby promoting neurovascular inflammation (Deane et al., 2003). In addition, an *in vitro* study showed that Aβ can increase the expression of RAGE, thus promoting a positive feedback loop (Wan et al., 2015). Importantly, the interaction of Aβ with RAGE was shown to trigger the disruption of tight junctions *via* the expression of MMP-2 and MMP-9 and *via* intracellular Ca^2 +^-calcineurin signaling (Kook et al., 2012).

The central nervous system was previously thought to be an immunologic sanctuary due to its BBB, acting independently in protecting the brain from disease. However, this ideology has been abandoned and the immune system is now considered an important player in AD pathology (Jevtic et al., 2017). Microglia are the main immune cells in the CNS. They are involved in its active surveillance and become activated upon brain injury or pathogen invasion. In the AD brain, microglia are activated around Aβ deposits inducing an innate immune response dominated by the release of pro-inflammatory cytokines and chemotactic factors that may act on peripheral immune cells. How these cells interact and contribute to AD pathology remains elusive. Although some are recruited to act locally in the brain, others may act from a distance (Wyatt-Johnson and Brutkiewicz, 2020). The initial microglia protective role in facilitating the clearance of Aβ results in uncontrolled neuroinflammation with chronic activation promoting the disintegration of the BBB (Zenaro et al., 2017).

Aβ deposition in the vasculature also leads to the release of proinflammatory cytokines and to oxidative stress that contributes to vascular dysfunction in AD. Aβ as well as its lipid carrier ApoE (encoded by the homonymous susceptibility gene) are vasculopathic, proinflammatory, impair Aβ degradation by microglial cells and astrocytes, impede efficient clearance and induce a degeneration of endothelial cells which is independent of other conditions (Kurz et al., 2021). Aβ peptides trigger vasoactivity activating pericytes and vascular smooth muscle cells further impairing BBB function (Fisher et al., 2022). For example, in a rodent model of AD, reactive oxygen species trigger the release of endothelin-1 that elicits pericyte contraction by acting on endothelin-A receptors, thus causing capillary constriction and reducing cerebral blood flow. Cumulative evidence suggests that vascular inflammation occurs during AD with higher levels of adhesion molecules associated with endothelial cell activation such as VCAM-1, ICAM-1, E-selectin, and P-selectin found in plasma samples from AD patients (Nielsen et al., 2007; Zuliani et al., 2008). A recent study contributed to the clarification of the role of vascular inflammation in BBB permeability (Propson et al., 2021). Briefly, activation of the signaling pathway involving the active peptide of complement component (C3a) and its receptor (C3aR) has been shown to modulate VCAM-1 expression, leading to an inflammatory transition in aged and transgenic mice. *In vitro* analyses identified Ca^2 +^ as a downstream effector of C3a/C3aR signaling and a functional mediator of vascular endothelial cadherin junction and barrier permeability.

Tau protein has also been showing an important role in regulation of the BBB. In physiological conditions, tau plays an important role in assembly and stabilization of microtubules, which are significant structural elements in cells of the CNS. In tauopathies like AD, tau undergoes different post-translational modifications, like hyperphosphorylation or truncation, altering its conformation, and aggregating in the form of insoluble neurofibrillary tangles in neurons and extracellular space. The effect of accumulated tau on BBB is mediated through the activation of glial cells which is associated with a pronounced inflammatory response, causing structural and functional changes. This process increases the formation of tau protein hyperphosphorylation, further promoting the formation of neurofibrillary tangles, giving rise to another deleterious feed-forward loop (Michalicova et al., 2017, 2020).

## 3. Blood-brain barrier as a target in Alzheimer’s disease

### 3.1. Potential molecular targets for blood-brain barrier targeting in Alzheimer’s disease

As previously mentioned, both influx and efflux Aβ transporters seem to be dysregulated in AD and have shown to be key players in Aβ accumulation. In this context, these transporters have been presented as potential therapeutic targets for AD. RAGE blockage have showed to slow down Aβ pathology and lower the rate of cognitive decline in animal models (Bell et al., 2012). However, the phase III clinical trial with a RAGE inhibitor (NCT02080364) was terminated due to lack of efficacy. Statins have been shown to upregulate LRP-1 at the BBB and reduce Aβ brain levels *in vitro* models. Although clinical trials have failed to show their efficacy, a recent reanalysis of these studies suggested that these drugs may benefit AD patients with potentially greater therapeutic efficacy in those homozygous for the APOE ε4 allele (Storck and Pietrzik, 2017), though it is not known whether the modulation of LRP-1 plays any role. *In vitro* studies support the role of P-gp in removing amyloid proteins (McCormick et al., 2021) and *in vivo* studies showed that prevention of P-gp degradation lowers Aβ brain levels (Hartz et al., 2018). However, to our knowledge, no LRP-1 or P-gp modulators have been clinically tested in AD yet.

The pathogenesis of AD has also been associated with a decreased expression of GLUT1, the major glucose transporter in the BBB. Liraglutide, a GLP1 analog, has showed to delay memory decline in a mouse model of AD (Hansen et al., 2015). Recently, it was demonstrated that the same drug slowed down memory decline in a group of patients with obesity and type 2 diabetes (Vadini et al., 2020) and more recently, GLP1 receptor agonists were shown to prevent glucose transport decline through BBB in AD patients, although no conclusions were drawn on cognitive decline (Gejl et al., 2016). Although little is known about the specific mechanism, it is thought that they could act by restoring the levels of GLUT1 at the BBB (Gejl et al., 2017). Also, a recent study pulling out data from randomized controlled trials and a nationwide cohort concluded that dementia incidence was reduced in patients with type 2 diabetes treated with GLP1 receptor agonists (Nørgaard et al., 2022). Based on these results, a phase IIb clinical trial (NCT01843075) demonstrated liraglutide improved cognitive function and MRI volume in patients with mild to moderate AD (Edison et al., 2021). Taken together, these data have been supporting GLP1 analogues as potential disease modifying therapy for AD. A phase III randomized controlled trial evaluating semaglutide in people with early AD is ongoing (NCT04777396).

**FIGURE 2:**
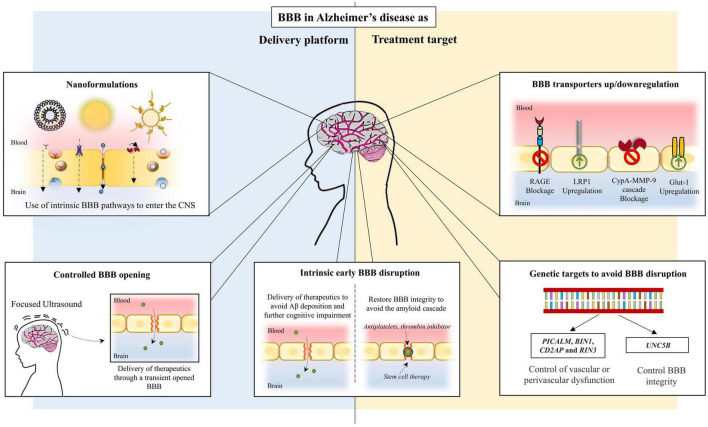
Conceptual blood-brain barrier targets in Alzheimer’s disease.

Lastly, inhibition of CypA-MMP-9 cascade, a pathway involved in BBB breakdown, in addition to repairing the BBB, also slowed down and reversed neurodegenerative changes in animal models (Bell et al., 2012). Table 1 presents BBB transporters/pathways implicated in AD and how they can be targeted. Figure 2 outlines BBB targeting strategies in AD.

**TABLE 1:** Blood-brain barrier targets in Alzheimer’s disease.

Target	Role	Regulation in AD	Therapeutic approach	Current status	References
LRP1	Aβ major efflux transporter across the BBB	Downregulated	Agonism/upregulation	*In vitro* studies with statins show upregulation of LRP1 at the BBB and consequently reduction of Aβ brain levels Reanalysis of clinical trials with statins indicates statins benefit, especially in APOE ε4 homozygous	Shinohara et al., 2010; Geifman et al., 2017
P-gp	Aβ efflux transporter across the BBB			*In vitro* studies support the role of P-gp in removing amyloid proteins *In vivo* studies show lower Aβ brain levels preventing P-gp degradation	Hartz et al., 2018; McCormick et al., 2021
RAGE	Aβ influx transporter across the BBB	Upregulated	Antagonism/blockage	Animal models show reduced brain Aβ levels and a lower rate of cognitive decline using either antagonists or anti-RAGE antibodies A phase III clinical trial of a RAGE inhibitor was terminated by lack of efficacy	Deane et al., 2012; Batkulwar et al., 2018
GLUT1	Glucose major transporter across the BBB	Downregulated	Agonism/upregulation	Animal models show GLP1 receptor agonists delay memory decline GLP1 receptor agonists slow down memory decline in patients with type 2 diabetes and prevent glucose transport decline through BBB in AD patients A phase IIb clinical trial evaluating a GLP1 analog demonstrated improved cognitive function and MRI volume A phase III clinical trial evaluating a GLP1 analog is ongoing	Hansen et al., 2015; Gejl et al., 2016; Vadini et al., 2020; Edison et al., 2021 EVOKE, NCT04777396
CypA-MMP9	BBB degrading pathway	Activated	Blockage	In animal models pharmacologic and genetic inhibition slow down and reverse neurodegenerative changes	Bell et al., 2012

AD, Alzheimer’s disease; LRP1, low-density lipoprotein receptor-related protein; Aβ, amyloid-β; BBB, blood-brain barrier; APOE, apolipoprotein E; P-gp, P-glycoprotein; RAGE, receptor for advanced glycation end products; GLUT1, glucose transporter 1; GLP1, glucagon-like peptide-1; CypA, cyclophilin A; MMP9, matrix metalloproteinase-9.

Despite positive outcomes from pre-clinical studies *in vitro* and *in vivo*, the scarcity of clinical trials emphasizes the necessity for additional research to bring these discoveries from the laboratory to the patient bedside.

### 3.2. Potential genetic targets for blood-brain barrier targeting in Alzheimer’s disease

Although the genetic underpins of BBB dysfunction are relatively understudied, genetic data has given a number of possible links between BBB dysfunction and AD. For instance, APOE ε4, the strongest genetic risk factor for AD (Farrer et al., 1997), has also been shown to lead to brain blood dysfunction and cognitive decline, independently of AD pathology (Montagne et al., 2020). This supports a link between BBB dysfunction and AD, but also suggests that BBB dysfunction may add cognitive decline independently. More recently, it has been shown that 30 of the top 45 genes that have been linked to AD risk by genome-wide association studies (GWAS) are expressed in the human brain vasculature, suggesting that vascular and perivascular systems are highly involved in AD pathogenesis (Yang et al., 2022). Genetic variants such as *PICALM*, *BIN1*, *CD2AP*, and *RIN3* have been linked to AD in GWAS and seem involved in BBB Aβ transcytosis pathways (Juul Rasmussen et al., 2019). A recent study has shown these variants to increase the risk of AD and vascular dementia, suggesting that there is a shared mechanism, possibly related to vascular or perivascular dysfunction (Juul Rasmussen et al., 2019).

Several monogenic mendelian diseases leading to brain disorders and cognitive impairment appear to originate in individual cell types of the neurovascular unit. Some of the genes are linked to specific roles in BBB development, function, and regulation (Zhao et al., 2015) but have also been linked with AD associated changes, such as *OCLN* (Romanitan et al., 2007), *COL4A1* (Pang et al., 2019; Xiao et al., 2021), *NOTCH3* (Sassi et al., 2018), *CSF1R* (Sassi et al., 2018), *TREM2* (Guerreiro et al., 2013), and *TYROBP* (Audrain et al., 2021). Furthermore, there is some evidence of BBB dysfunction happening in a number of genetically determined degenerative disorders, such as AD, but also Parkinson’s disease, Huntington’s disease and amyotrophic lateral sclerosis (Katt et al., 2019).

Other studies such as those enumerated in Table 2 have identified other potential genetic links between BBB regulation and development and AD pathogenesis, providing additional potential drug targets. For example, GLUT1 is the most important energy carrier of the brain across the BBB, and shown to be critical in BBB development (Zheng et al., 2010). It has been reported that GLUT1 reductions exacerbate AD pathology (Winkler et al., 2015). Although the syndromes currently associated with *GLUT1* pathogenic variants start at a very young age (Koch and Weber, 2019), small-effect changes with low frequency in the population can potentially be associated with the risk of developing AD. This is further supported by some genetic data in drosophila models (Shulman et al., 2011). Likewise, MFSD2A, another major transporter with a key role in modulating transcytosis to regulate the BBB (Andreone et al., 2017), expression is reduced in AD patients (Sánchez-Campillo et al., 2019).

**TABLE 2:** Genetic links between Alzheimer’s disease and/or cognition and blood-brain barrier dysfunction.

Gene	Role/Link	References
APOE ε4	Both the strongest genetic risk factor of AD and associated with BBB dysfunction irrespective of AD pathology	Farrer et al., 1997; Montagne et al., 2020
*PICALM*, *BIN1*, *CD2AP* and *RIN3*	BBB Aβ transcytosis pathways and associated with AD in GWAS	Juul Rasmussen et al., 2019
*OCLN*	Occludin overexpression in AD and VD	Romanitan et al., 2007
*COL4A1*	Component of basement membrane whose defect may lead to cortical malformations through vascular damage	Pang et al., 2019; Xiao et al., 2021
*NOTCH3*	Dysfunction transmembrane receptor expressed in vascular smooth muscle cells and pericytes that may promote AD susceptibility by increasing the risk for small vessel disease or leukoencephalopathy.	Sassi et al., 2018
*CSF1R*	Colony stimulating factor that regulates macrophages, enhances BBB permeability and is overexpressed in cortex with Aβ pathology	Sassi et al., 2018
*TREM2*	Triggering receptor expressed on myeloid cells 2 protein expressed in microglia correlated with BBB integrity and whose variants increase the risk of AD	Guerreiro et al., 2013
*TYROBP*	Cytoplasmic adaptor for receptors such as TREM2 that influences microglia – a key component of neurovascular unit – to become diseased and may drive AD	Audrain et al., 2021
*GLUT1*	Glucose transporter across BBB crucial in its development and reduced in AD	Zheng et al., 2010
MFSD2A	BBB transporter that modulates transcytosis and is underexpressed in AD	Andreone et al., 2017; Sánchez-Campillo et al., 2019
*PRF1*	BBB transporter linked with Aβ processing and its internalization in neurons	Willenbring et al., 2016; Lana et al., 2017
*FML2*	Protein that regulates glia and vasculature interaction and is overexpressed in AD and in cerebrovascular pathology	Lee et al., 2022
*UNC5B*	Netrin receptor that regulates angiogenesis, controls BBB integrity and the same gene family of *UNC5C* whose mutations predispose to AD	Boyé et al., 2022
*FOXO1*	Transcription factor that influences BBB changes, Aβ production and tau phosphorylation	Zhang et al., 2020

AD, Alzheimer’s disease; BBB, blood-brain barrier; Aβ, amyloid-β; GWAS, genome-wide association studies; VD, vascular dementia.

A GWAS (Lee et al., 2022) has looked into FML2, a formin-related protein expressed in astroglial cells that regulares glia and vasculature interaction and partakes in the homeostasis and clearance of amyloid β. It has shown that FML2 is overexpressed in AD and in cerebrovascular pathology independently. Expression of FMNL2 increases in the presence of vascular risk factors among individuals with AD, and it appears that this gene plays a significant role in the progression of AD pathology when both conditions are present.

Several other transporters may also link both disorders. *PRF1*, for example, which has been linked with BBB dysfunction (Willenbring et al., 2016), has also been linked with amyloid processing, being shown to promote amyloid-beta internalization in neurons (Lana et al., 2017). Recently, *UNC5B* has been suggested to control BBB integrity (Boyé et al., 2022). This gene is of the same family of *UNC5C*, which has been repeatedly linked with AD. Another link between AD and BBB dysfunction comes from a model of multiple sclerosis, where *FOXO1* downregulation has also been linked to BBB changes (Mora et al., 2020). Interestingly, *FOXO1* upregulation has been shown to reduce Aβ production and tau phosphorylation *in vitro* (Zhang et al., 2020).

This data gives support to the idea that BBB dysfunction is an important step in AD pathophysiology, possibly by facilitating known mechanisms but perhaps also by adding additional damage to AD patients’ brains by other mechanisms.

## 4. Blood-brain barrier as a therapeutic vehicle in Alzheimer’s disease

One of the major limitations for the development of AD treatments, as for the vast majority of CNS disorders, is the design of a system able to penetrate the selective nature of the BBB (Srivastava et al., 2021). As already described in this report, the BBB is highly sealed and selective and hence avoids the entrance of unwanted compounds into the brain (Bernardo-Castro et al., 2020) therefore, finding a way to overcome it is a key for AD treatment.

Despite its efficient barrier function, there are several transport routes that allow a selective exchange of compounds through the BBB (Bernardo-Castro et al., 2020). Taking advantage of these intrinsic mechanisms will permit the delivery of the desired therapeutic compounds into the brain. Nonetheless, one of the major problems of therapeutic compounds designed to cross the BBB is that they are unable to reach the key targets in the brain without losing bioavailability, solubility, stability or efficacy. To avoid this, the most highlighted strategy to improve AD treatment is the use of nanomaterials able to enhance the permeation of therapeutic compounds through the BBB to reach the desired target without losing its properties (Alphonsus and Rodseth, 2014). As such, the highly selective feature of the BBB may be exploited as a therapeutic vehicle to specifically deliver therapeutic compounds that directly target AD pathological features. Figure 2 summarizes pathways of BBB delivery in AD.

### 4.1. Nanoformulations for Alzheimer’s disease

Nanomaterials for targeted drug delivery across the BBB have been extensively studied in the medicine field, particularly in AD (Faiyaz et al., 2021). In order to cross effectively the BBB and target directly the AD brain, nanoformulations should have: stability in blood circulation, proper surface modification, amyloid direct targeting that does not interfere with BBB targeting (multifunctionality) and ability to release at the target site (Tosi et al., 2019).

A vast variety of nanotechnology-based approaches fulfilling these characteristics have been and are currently being adapted to surpass the BBB in AD (Ordóñez-Gutiérrez and Wandosell, 2020; Srivastava et al., 2021; Chopra et al., 2022). Some relevant examples of AD nanoformulations include lipoprotein-based, polymers or metallic nanoparticles (Karthivashan et al., 2018; Bilal et al., 2020; Faiyaz et al., 2021).

The rationale behind these nanotechnology-based approaches is to take advantage of the intrinsic transport strategies of the BBB to efficiently reach brain tissue and target AD’s pathological processes. For instance, lipophilic nanoparticles directed toward the brain endothelial cells (BECs) could allow the transport of therapeutic compounds through endocytosis or lipophilic transcellular pathways (Karthivashan et al., 2018). In this context, solid lipid nanoparticles loaded with curcumin (a potent antioxidant, anti-inflammatory compound with anti-Aβ accumulation effects) have shown to efficiently surpass the BBB due to their lipidic nature and to reduce oxidative stress in the hippocampal tissue improving spatial memory in an AD rodent model (Sadegh Malvajerd et al., 2019). Other strategies such as ionized-nanomaterials could take advantage of the negatively charged BECs membrane to surpass the BBB through adsorptive transcytosis. For example, the conjugation of deferasirox (an iron-chelating agent) to cationized human serum albumin showed correct brain uptake though adsorptive transcytosis with attenuated amyloid beta-induced learning deficits in a rat model of AD (Kamalinia et al., 2015). Another strategy comprises functionalized nanomateriales including liposomes, polymeric or metallic nanocarriers. This strategy relies on receptor-mediated transcytosis and carrier protein-mediated pathways taking advantage of the already existing receptors in the cells surface to transport the therapeutic cargo across the BBB (Karthivashan et al., 2018). A good example of this mechanism is the use of LPR-1 as delivery system across the BBB. Polymeric nanoparticles functionalized with Angiopep-2 (a specific ligand to LPR-1) and loaded with Prussian blue showed efficient BBB crossing and restoration of mitochondrial function along with reduced neurotoxic Aβ aggregation (Zhong et al., 2022). A gold standard in receptor mediated transcytosis is the transferrin receptor (TfR). The TfR is highly expressed in the BBB and has been prove to be functional in the BBB during AD (Bourassa et al., 2019), hence conforming a great candidate for brain delivery in AD. In this context, transferrin-functionalized liposomes loaded with gallic acid where proven to efficiently bypass the BBB through TfR-mediated transcytosis and efficiently decrease the number of Aβ fibrils formed by 56% (Andrade et al., 2022).

Despite all, none of these strategies aiming to a single AD process has achieved the expected results in terms of disease treatment. To this aim, and due to the complex nature of AD and the multifactorial regulation of Aβ aggregation, multitarget nanotherapeutics seem to be a promising strategy to target not only this hallmark but also complementary pathways involved in this disease (Ibrahim and Gabr, 2019). In this context, self-assembled Fmoc-Trp-Fe^2 +^ -Que NPs have shown successful suppression of Aβ plaques, reduction of reactive oxygen species (ROS) generation and suppression of the neurotoxicity induced by Aβ (Zhu et al., 2022). Furthermore, chondroitin sulfate-selenium NPs have also shown to be very promising as multitarget AD therapy showing reduction in ROS levels and cytoskeleton damage, attenuation of hyperphosphorylation of tau and Aβ aggregation inhibition (Gao et al., 2020).

All in all, nanoformulations present themselves as one of the most promising tools for AD treatment, taking advantage of the selective BBB characteristics and using it as a delivery platform to selectively target the brain. Nonetheless, albeit very promising, its use is limited due to safety concerns and in most cases failure to achieve adequate concentrations of the delivered compounds to the brain tissue (Lipsman et al., 2018). Moreover, BBB transport mechanisms can be altered during neurodegeneration which will, in turn, affect the delivery of therapeutic compounds and hamper the use of this mechanism as a delivery platform (Wasielewska and White, 2022).

In this context, controlled opening of the BBB has been brought up as an alternative potential mechanism to facilitate paracellular drug delivery into the brain using the BBB as a platform.

### 4.2. Controlled BBB permeability as a therapeutic vehicle

Controlled BBB opening should be transient and selective to avoid unwanted accumulation in the brain and any potential side effects (Han, 2021). Several pre-clinical and clinical studies have studied this controlled opening of the BBB as a way to the brain in the treatment of AD. For instance, mannitol has been used *in vitro* to reversibly open the BBB (Haluska and Anthony, 2004). Nonetheless, focused ultrasound in combination with microbubbles currently constitutes the only truly transient, localized, and non-invasive technique for opening the BBB (Konofagou et al., 2012). Significant progress has been made in the pre-clinical validation and development of focused ultrasound which eventually lead to the initiation of clinical trials examining its application for delivery in AD (Wasielewska and White, 2022). In fact, this technique has been shown to reversibly open BBB in AD patients. A clinical trial investigated BBB opening in the white matter of the superficial dorsolateral prefrontal cortex of AD patients demonstrating that this opening is safe in humans (Lipsman et al., 2018). Hippocampal BBB opening has been also demonstrated safely in a proof-of-concept study that also reported an Aβ decrease after opening (Rezai et al., 2020). In this line, a recent phase I/II trial in early stage AD showed that repeatedly opening the BBB with ultrasound is well-tolerated and may be associated with a reduction of amyloid burden (Epelbaum et al., 2022). Extensive opening of the BBB (above 20 cm^3^) has also been shown safe and potentially beneficial with a potential anti-amyloid effect by itself (Park et al., 2021).

While the potential benefits of BBB opening by itself are still not clear, the proof of safety in transiently opening the BBB of AD patients could additionally allow the delivery of larger molecules such as antibodies or growth factors (Lipsman et al., 2018) that directly target the AD pathology taking advantage of the increased permeability. Nonetheless, BBB opening raised several concerns such as potential brain toxicity due to the non-specific accumulation of neurotoxic substances from the blood that could produce neuronal damage and degenerative changes (Han, 2021).

Interestingly, BBB disruption has been reported to occur in AD even before the onset of hippocampal atrophy (Montagne et al., 2015; Sweeney et al., 2018) and hence the evidence of a naturally permeable BBB in the early phases of the disease could allow the delivery of therapeutic compounds even at the early stages of AD without invasive measures.

### 4.3. Integrating blood-brain barrier in the therapeutic landscape of Alzheimer’s disease

Currently, no disease-modifying therapies for AD are approved by the European Medicines Agency (EMA). In the USA, Aducanumab (Aduhelm™) has been given Food and Drug Agency (FDA) approval under the agency’s accelerated approval pathway for AD treatment, meaning a post-approval trial has to demonstrate clinical benefit to keep the approval. Aducanumab, a human IgG1 anti-Aβ monoclonal antibody selective for Aβ aggregates, has become the first FDA-approved drug to reduce Aβ levels, a decision that was not devoid of criticism among the scientific community (Tampi et al., 2021). More recently, another accelerated approval has been granted by FDA to Lecanemab (Leqembi™) after the results of an 18-month, phase 3 trial that involved 1795 patients with early AD. This humanized IgG1 monoclonal antibody which selectively binds to large, soluble Aβ protofibrils has shown a reduction of Aβ in early AD patients and less decline in measures of cognition and function with modest effect sizes (van Dyck et al., 2022; Gandy and Ehrlich, 2023). Other Aβ-targeted monoclonal antibody therapies have failed to show positive clinical outcomes. Several reasons have been pointed out for such failure one being poor antibody brain penetration (< 0.1%) (van Dyck, 2018; Bajracharya et al., 2021; Nehra et al., 2022). In fact, it was only in the high dose intervention arm of the Aducanumab EMERGE trial that clinical benefit was found, suggesting the need of higher accumulated doses of the antibody to compensate for the low penetrance (Schneider, 2020; Leinenga et al., 2021). As pointed above, ultrasound has the potential to open BBB (Leinenga et al., 2016) constituting an option to alternatively enhance the clearance of amyloid by facilitating para and transcellular transport across this barrier (Pandit et al., 2020) and to facilitate drug delivery to the central nervous system. That is why, just recently, Leinenga et al. (2021) have investigated separately Aducanumab and scanning ultrasound in an animal model, showing that each one of these strategies has a comparable potential to reduce plaque burden. This suggests that the latter may be a treatment option in AD and hypothesizes that both should be studied in a combination trial as an approach to increase the brain levels of Aducanumab and to treat AD. As mentioned in a study cited above, magnetic resonance-guided focused ultrasound was able to open transiently and non-invasively the BBB in 5 patients with Aβ AD (Lipsman et al., 2018). A non-eloquent brain region was targeted and this technique was not complemented with any treatment which may soon be tested.

Other strategies to increase the delivery of drugs across BBB, particularly monoclonal antibodies, that are currently under development include bi-specific antibodies (a group that included the above-mentioned LRP-1 and other receptor-mediated transporters), nanoparticles (including liposomes, metallic nanoparticles and dendrimers), exosomes, viral vectors (Bajracharya et al., 2021)and drug re-engineering as fusion proteins that interact with BBB transcytosis systems, particularly transferrin receptor monoclonal antibodies (Pardridge, 2015). This latter strategy has been coined Brain Shuttle-mAb technology which has been shown to target Aβ in a mouse model of AD 55-fold compared to the parent antibody and significantly improves Aβ reduction (Niewoehner et al., 2014).

A pitfall associated with immunotherapy against Aβ in AD has been the adverse effects, particularly Aβ-related imaging abnormalities (ARIA) such as cerebral edema (ARIA-E) and microhemorrhages (ARIA-H). These indicate a state of increased barrier permeability and leakage in these patients (Nehra et al., 2022) also observed spontaneously in patients with cerebral amyloid angiopathy. This means that BBB permeability is a double-edged sword: on the one hand, it represents a barrier needed to be crossed over for efficacious delivery of immunotherapy and on the other hand, its dysfunction may precipitate the occurrence of adverse effects associated with such immunotherapy.

Research on disease modifying therapies in Alzheimer’s has been focused on Aβ, as it is the most common target of phase 2 and phase 3 clinical trials (Scheltens et al., 2021). However, anti-tau therapies have also been gaining momentum through phase 1 and 2 trials (Cummings et al., 2019). Similar to anti-amyloid antibodies, focused ultrasound has been used as an effective strategy to enhance the delivery of anti-tau antibodies in AD models (Jordão et al., 2010; Nisbet et al., 2017; Janowicz et al., 2019; Xhima et al., 2020).

Targeting the BBB may be a therapeutic strategy in itself. A leaky BBB is part of the pathophysiological conundrum of AD. Either as a consequence of inflammation and Aβ-induced cerebral amyloid angiopathy or part of the AD pathophysiological cascade that results in neurodegeneration and dementia (Noe et al., 2020). Therefore, drugs that repair BBB represent a window of opportunities. In pre-clinical AD models, thrombin inhibitor dabigatran (Cortes-Canteli et al., 2019), antiplatelet agents such as dipyridamole, cilostazol, tadalafil (Fisher et al., 2011; García-Barroso et al., 2013; Hattori et al., 2016) and recombinant activated protein C (Lazic et al., 2019) have shown barrier-sealing effects (Nehra et al., 2022). Another class of drugs that may prevent barrier dysfunction is angiotensin-II receptor blockers, particularly olmesartan. It has been demonstrated to act on tight junction mRNA levels and limit oxidative stress in mice (Nakagawa et al., 2017a,b).

Stem cell therapy has also the potential to target BBB. Stem cells can be differentiated into mesenchymal stem cells, neural stem cells, and induced pluripotent stem cells. All of these have been studied in AD with several purposes beyond BBB repair. The most used has been mesenchymal stem cells (Kim et al., 2020). To repair BBB, both mesenchymal stem cells and induced pluripotent stem cells both have the potential to differentiate into endothelial cells and regenerate blood vessels. In an *APP/PS1* mouse model, bone marrow mesenchymal stem cells—VEGF treatment improved endothelial dysfunction, neovascularization and reduced senile plaques in the hippocampus with impact on cognitive dysfunction of AD transgenic animals (Garcia et al., 2014). Another lineage of stem cells that has been studied in BBB dysfunction related to AD is endothelial progenitor cells. They derive from the bone marrow and are thought to represent the main cell lineage involved in endothelial repair mechanisms (Custodia et al., 2021). In an APP/PS1 mouse model, endothelial progenitor cells transplantation has been shown to repair BBB tight junction function, increase microvessel density and decrease Aβ senile plaque deposition (Zhang et al., 2018).

## 5. Future perspectives

We still do not know today the complete pathophysiology of AD. The shortcomings of recent clinical trials evaluating anti-Aβ therapies prove that. Accumulating evidence points to a strong link between BBB dysregulation and AD as we have previously demonstrated. Even so, the exact role of BBB dysfunction in the overall pathogenic cascades of AD has not yet been determined. Therefore, one major goal for future and ongoing basic and translational research is to improve our general understanding of the pathophysiological pathways underlying AD and the role played by BBB dysfunction. The hope is that a deeper understanding of how BBB is affected in early AD will lead to the identification of novel and efficacious targets to prevent or repair neurovascular dysfunction thereby slowing or stopping AD progression.

Regarding clinical research, efforts should be put in targeting BBB to: (1) increase the delivery of CNS therapeutic substances overcoming the selectiveness of its nature and the low cerebral bioavailability of drugs administered by peripheral routes; (2) avoid BBB drug-induced dysfunction, particularly the adverse effects associated with immunotherapy such as ARIA-E and ARIA-H both secondary to BBB damage; (3) repair and enhance BBB clearance mechanisms by known transporters such as LRP-1 and RAGE reducing Aβ and another toxic burden.

## 6. Conclusion

Blood-brain barrier dysfunction is an early event in AD, a burdensome disease that is lacking disease-modifying therapies. Several common molecular and genetic changes have been found to link vascular dysfunction and AD. The delivery of drugs across the central nervous system has long been and still is an obstacle. In both premises, BBB plays a key role either by representing a potential therapeutic target or a therapeutic vehicle.

## Author contributions

JS and CB drafted the manuscript with the contributions from SB-C, MT-P, ML, and IA. JS-F was responsible for the manuscript conception and supervision. IB, LF, JB, RG, and IS reviewed the scientific content. All authors read and approved the final manuscript.
